# Detection of Relative Afferent Pupillary Defects Using Eye Tracking and a VR Headset

**DOI:** 10.1167/tvst.12.6.22

**Published:** 2023-06-27

**Authors:** Dominik Bruegger, Hilary M. Grabe, Rino Vicini, Muriel Dysli, David Lussi, Mathias Abegg

**Affiliations:** 1 Department of Ophthalmology, Inselspital, Bern University Hospital, University of Bern, Bern, Switzerland; 2 Graduate School for Cellular and Biomedical Sciences (GCB), University of Bern, Bern, Switzerland

**Keywords:** relative afferent pupillary defect (RAPD), eye tracking, virtual reality (VR) headset

## Abstract

**Purpose:**

The purpose of this study was to assess the feasibility of detecting relative afferent pupillary defects (RAPDs) using a commercial virtual reality headset equipped with an eye tracker.

**Methods:**

This is a cross-sectional study in which we compare the new computerized RAPD test with the traditional clinical standard using the swinging flashlight test. Eighty-two participants including 20 healthy volunteers aged 10 to 88 years were enrolled in this study. We present a bright/dark stimulus alternating between the eyes every 3 seconds using a virtual reality headset, and we simultaneously record changes in pupil size. To determine the presence of an RAPD, we developed an algorithm analyzing the pupil size differences. For the assessment of the performance of the automated and the manual measurement a post hoc impression based on all available data is created. The accuracy of the manual clinical evaluation and the computerized method is compared using confusion matrices and the gold standard of the post hoc impression. The latter is based on all available clinical information.

**Results:**

We found that the computerized method detected RAPD with a sensitivity of 90.2% and an accuracy of 84.4%, as compared to the post hoc impression. This was not significantly different from the clinical evaluation with a sensitivity of 89.1% and an accuracy of 88.3%.

**Conclusions:**

The presented method offers an accurate, easy to use, and fast method to measure an RAPD. In contrast to today's clinical practice, the measures are quantitative and objective.

**Translational Relevance:**

Computerized testing of Relative Afferent Pupillary Defects (RAPD) using a VR-headset and eye-tracking reaches non-inferior performance compared with senior neuro-ophthalmologists.

## Introduction

The assessment of the relative afferent pupillary deficit (RAPD) is one of the three cornerstones on which the clinical evaluation of the afferent visual system is based. Together with reliable measures of visual acuity and visual field, the RAPD allows one to determine the location of any lesion within the afferent visual pathways.^1^

In contrast to determining visual acuity and the visual field (perimetry), the assessment of an RAPD may be done within a few seconds. Today, it is usually measured using the swinging flashlight test as named by Levatin et al.^2^ and made popular by Thompson.^3^ Some specialists use neutral density filters which allow a quantitative measure in log units and a higher sensitivity and specificity as compared to the swinging flashlight test alone.^4^ However, the use of neutral density filters requires practice and is usually reserved for more specialized clinicians.

As used in clinical settings today, the assessment of afferent pupillary defects is associated with several problems. (1) It usually is determined qualitatively rather than quantitatively thus often preventing judgment about disease progression. (2) It is usually measured manually, and thus critically depends on the skills of the examiner. (3) As measured in today's clinical practice it is unreliable: Boucher et al. found that only about 75% of RAPDs are correctly identified and the magnitude of an RAPD was estimated correctly in about 40%.^5^ Additionally, Hennessy et al. showed that non-experts only found 12% of patients with an RAPD; this number was much higher when measured with well-trained experts.^6^

It has long been recognized that these problems could be mostly resolved with a video-based objective measure of the pupil size over time (pupillography) combined with the application of a light stimulus to either eye. The basic principles of such quantitative pupillography along with a description of how to analyze pupillary movement to determine an RAPD were proposed as early as 1954 by Lowenstein.^7^ Since then, several groups have refined testing methods, which resulted in an excellent accuracy of objective measures in contrast to human measures. For example, Volpe and colleagues attained a 91% sensitivity with a 95% specificity to detect an RAPD larger or equal than 0.5 log units measured with a neutral density filter.^8^^,^^9^ Today, some commercial products are available that measure an RAPD (e.g. RAPIDO; Neuroptics Inc. USA, and EyeKinetix; Konan Medical USA, Inc., USA) with similar protocols and probably similar efficacy. However, these devices are usually used in a research context or at specialized clinics with a particular focus on neuro-ophthalmology and thus advanced clinical skills in RAPD assessment. Conversely, the greatest clinical need is in medical areas with less expertise: general ophthalmologists, optometrists, or general practitioners. Practitioners who only occasionally must identify or exclude an RAPD are likely discouraged from buying an expensive medical device for financial reasons.

Here, we investigate the use of a virtual reality (VR) display with an integrated eye tracker for the clinical purpose of measuring an RAPD. VR headsets are commonly used in consumer electronics and are increasingly being used in medical applications.^10^ Although we used the same VR headset for all measurements in our study, a number of VR systems are available with eye tracking and the software could be used with different systems.^11^ This may represent a cheap and readily available tool for interested health practitioners.

## Materials and Methods

### Study Design

Participants were recruited from the neuro-ophthalmology service of an outpatient clinic at a tertiary care center (Inselspital, University Hospital Bern, Bern, Switzerland). Clinical examination was performed by an experienced neuro-ophthalmologist (authors M.A. or H.G). We asked all patients that were over 18 years old and who had an RAPD as determined with the swinging flashlight test during the clinical routine to participate in the study. We included borderline cases in which clinicians were uncertain about the result of the swinging flashlight test despite using neutral density (ND) filters. We quantified the RAPD using neutral density filters for a subset of the patients. For this, we repeated the swinging flashlight test with ND filters of different strengths (0.3, 0.6, 0.9, 1.2, 1.5, and 1.8 logUnits) in front of the healthy eye until the RAPD disappeared or even changed the side. The density of the ND filter which neutralizes the RAPD determines the magnitude of the RAPD. We have included patients with unilateral or asymmetric optic neuropathies, such as optic neuritis, ischemic, or compressive optic neuropathies, in this study. Additionally, we included 18 participants with no known eye disease as controls. All participants provided written informed consent; the local ethics committee (Kantonale Ethikkomision Bern [KEK], Basec PB_2016-00250) approved all study procedures, which were performed in accordance with the principles of the Declaration of Helsinki.^12^

### Optic Neuropathy Versus Swinging Flashlight Test

We developed a post hoc impression for all patients to determine whether a nerve damaging disease creating an RAPD should have been present, based on all available data. This post hoc impression is based on all available clinical data, including visual acuity, color vision, visual field examination, fundoscopy, and peripapillary nerve fiber layer thickness measures with optical coherence tomography. Cases with unclear or uncertain diagnosis were excluded from further analysis. The examiners were not blinded to the history, known diagnoses, or clinical findings of the patients.

### Test Setup

**Figure 1.**
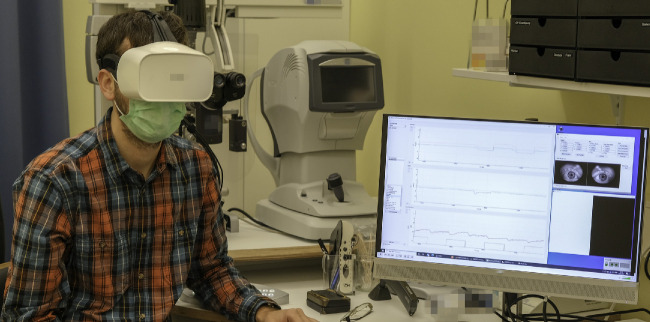
Photograph of a participant wearing the VR headset, and the output on the screen during the examination, showing the Image feed from the IR-eye tracking cameras used for alignment and compliance monitoring. Beside the camera feed the graphical user interface of the custom developed software for data visualization and recording is shown.

All study measurements were performed with the Fove DK0 (Fove Inc., Japan), a commercially available VR headset equipped with a binocular eye tracker. Figure 1 shows a participant wearing the headset and the output of the near infrared (IR) cameras. We used the Unity3D engine (Unity Technologies, San Francisco, CA, USA) to develop and present the repetitions of alternating bright and dark stimuli. The eye tracker data were recorded and visualized in real-time using a custom developed program written in C++. Pupillary and gaze data are sampled and saved for both eyes at a rate of 120 Hz. Visual stimuli are presented on a WQHD OLED screen run at a brightness of 120 cd/m^2^ using a pulse width modulation (PWM) duty cycle of 30.7%, and a screen refresh rate of 70 Hz.

### RAPD Testing Procedure

**Figure 2.**
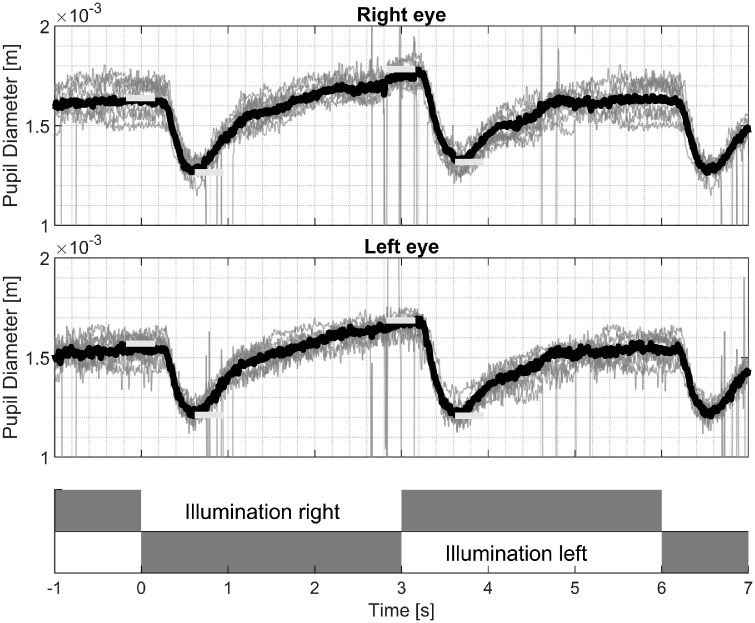
Top two plots: Time-synchronized pupil size recordings of multiple light switches between the left and right eyes from one patient with a right sided RAPD. The *top panel* shows recordings from the right eye; the *middle panel* corresponds to the left eye. *Grey* = single recordings; *black* = averaged traces with outliers removed; *horizontal bars* = amplitude values for further processing. *Bottom panel* = Illumination sequence for the right and left eyes, with illuminated = white, and dark = grey.

The VR headset is positioned on the head of the participant, and its alignment is checked and adjusted using the video-stream of the built in IR cameras. After running the device-specific calibration sequence, we presented an alternating bright/dark stimulus by setting all pixels on one side to either white or black, and alternated between the sides every 3 seconds (see Fig. 2) while we recorded the pupil size. The change of bright stimulus from one eye to the other was instantaneous without a pause in between the changes. We instructed the participants to look straight ahead, and they were allowed to blink normally. We presented the alternating bright/dark stimuli for approximately 1 minute and prolonged the duration in cases where the examiner felt that longer testing was helpful, primarily in cases with increased noise levels in the recordings and due to excessive blinking.

### RAPD Marker Extraction

We used the correlation between the values that are one sample apart (lag 1 autocorrelation) as a quality criterion to detect recordings with excessive amounts of noise. The recordings from both eyes are processed independently, and the data for the eye(s) which do not reach the quality threshold of 0.95 are removed from further analysis.

To account for different surrounding conditions before setting up the headset and reduce variation over the time course of the acquisition, we remove the first stimulus from further calculations. For the determination of an RAPD, we calculate the average over the remaining repetitions independently for both eyes. Blinking led to a transient loss of pupil size measure and hence a loss of data during the time of a blink and we calculated the average using the remaining data. We then extract the maximal pupil size before the side change of the stimulus and the minimal pupil size just after the switch. To search for those values, we use 333.33 ms long sections, as visualized in Figure 2 with bold bars.

With the extracted values for maximal and minimal pupil size we calculate two features for further processing:

*Pre-stimulus amplitude difference* describes the difference of the maximal pupil size between right and left side illumination (see Fig. 2).

(1) $$\begin{array}{ccc} & & \mathrm{Pre}-\mathrm{Stimulus}\mathrm{Amplitude}\mathrm{Difference}\\ & & \;=P_{maxRight}-P_{maxLeft}\; \end{array}$$

*Contraction amplitude difference* describes the difference between the constriction amplitudes for the left and right side’s illumination.

(2) $$\begin{array}{ccc} & & \mathrm{Contraction}\mathrm{Amplitude}\mathrm{Difference}\\ & & \;=\left(P_{maxRight}-P_{minRight}\right)\\ & & \;-\left(P_{maxLeft}-P_{minLeft}\right)\; \end{array}$$

An example of a right sided RAPD is shown in Figure 2.

As both eyes do not show the exact same pupillary reaction with monocular stimulation (contraction anisocoria, see below), we averaged the extracted features of both eyes if the quality criterion is fulfilled for both. Only if one side does not fulfill the quality criterion, the recording from the other side is used for further processing.

We normalize the extracted features by division through the maximal pupillary size to decrease the influence of different pupil sizes.

**Figure 3.**
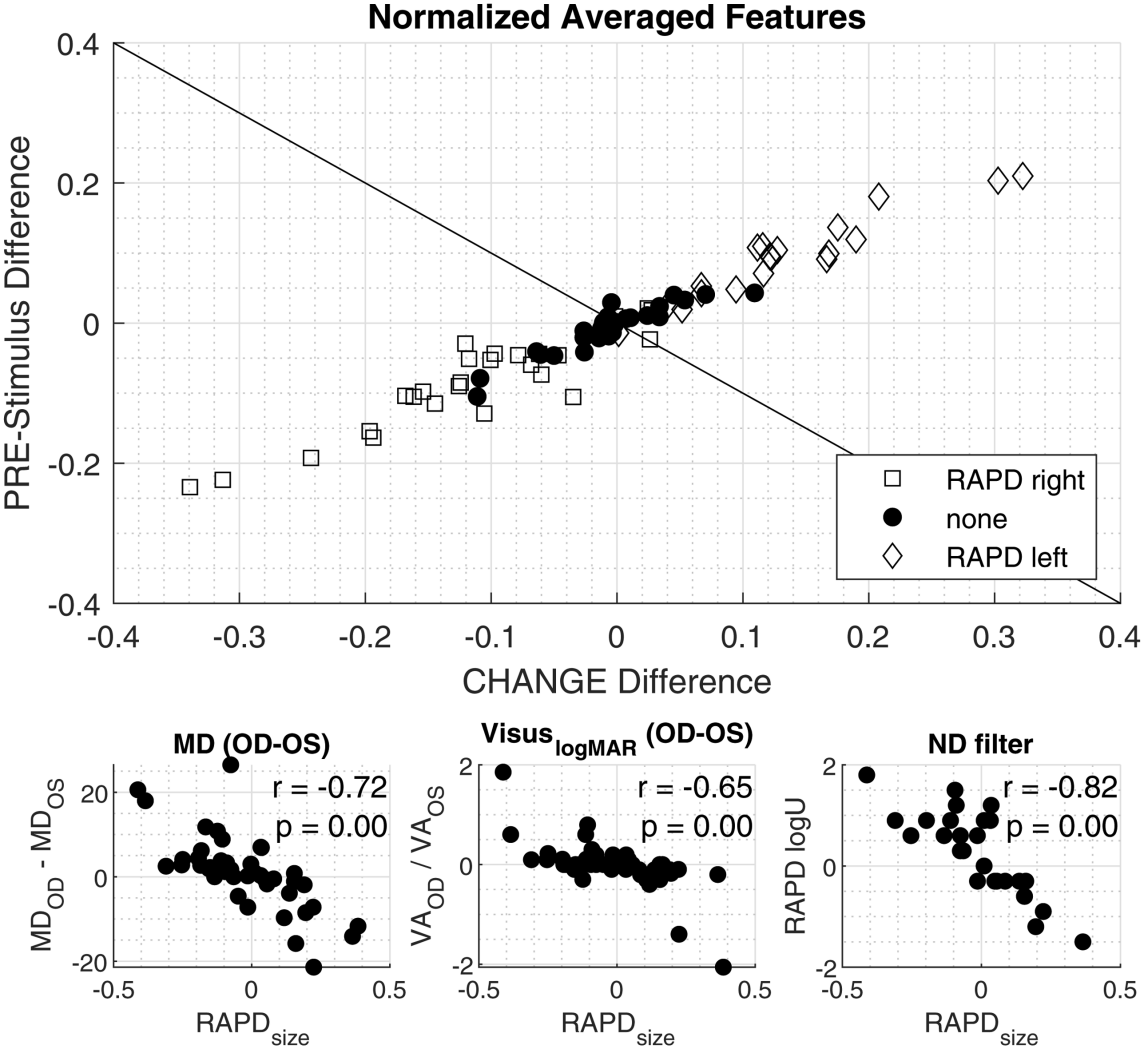
*Top*: Scatter plot shows the pre-stimulus amplitude difference (i.e. the difference of maximal dilation with left sided illumination and right sided illumination on the y-axis). The x-axis shows the difference of pupillary amplitude with right sided illumination and left sided illumination. Positive RAPD values correspond to a left sided RAPD, whereas negative values indicate a right sided RAPD. The *tilted line* separates the left and right sided RAPD. *Bottom-left*: Correlation between mean defect of the visual field and RAPDSize (for definition see Methods). *Bottom-center*: Correlation between RAPDSize and the right- and left difference in logMAR visual acuity. *Bottom-right*: Correlation between RAPDSize and the required neutral density (ND) filter to neutralize the RAPD in the swinging flashlight test. Positive RAPD values correspond to a left sided RAPD, while negative values indicate a right sided RAPD.

We then combine the two measurements into one by viewing it as a complex number with pre-stimulus amplitude difference being the real part and contraction amplitude difference the imaginary part, and name its absolute value RAPDSize this means that RAPDSize is defined as the root mean square of pre-stimulus amplitude difference and contraction amplitude difference*.* We define the RAPDSize as negative when its angle lies in the range between 3/4 π and −1/2 π, with negative values used to indicate a right RAPD. The separation of the right and left RAPDs is indicated with a slanted line in the top-panel of Figure 3.

To calculate the threshold values for the automated classification of an RAPD, we performed receiver operating characteristic analysis based on RAPDSize and the post hoc impression. We optimized the threshold for maximal accuracy.

Signal processing and statistical evaluation is done using Matlab R2020a (The MathWorks Inc. Natick, MA, USA), and R version 3.63.

### Evaluations

We checked the validity of our RAPDsize feature by comparing it to established clinical features, such as neutral density filters that may be used to quantify an RAPD, mean defect (MD) size in the visual field test using the Haag-Streit Octopus 900, and visual acuity measurements.

To examine the influence of contraction anisocoria,^13^ which is defined by a bigger contraction in the directly illuminated pupil than the contralateral pupil, we analyzed the size difference of the pupil size for direct versus contralateral illumination.

## Results

Pupillography was performed in 82 test participants. Three participants were excluded (2 unclear clinical cases and one due to data loss), of the remaining 79 datasets, 12 eyes of 10 participants did not fulfill the defined lag 1 autocorrelation-based quality criterion. Data from a total of 77 participants (binocular datasets of 47 patients and 20 healthy volunteers, and 10 monocular datasets of patients) were included in the final analysis. Median number of dark-bright stimulus repetitions was 10 (range = 4–29). Using an i1Display Pro (X-Rite Inc.) we measured a brightness of 102.040 ± 0.513 cd/m2 for the bright stimulus and 0.011 ±0.001 cd/m2 for the dark stimulus at the level of the lens of the headset.

In Table 1, we summarize the performance of our test as compared with the post-hoc reference. Our automatic VR headset-based method detected an RAPD with an accuracy of 84.4% (see Table 1), compared with the accuracy of 88.3% of the manual swinging flashlight test (see Table 2). We found no significant difference between the two methods using McNemar^13^ test (*χ*^2^ = 0.75, *P* = 0.39). We tested the same automatic classification method using the averaged data from binocular measurements also with monocular measurements achieving the following accuracy: Binocular = 84.4%, OD = 77.0%, and OS = 81.9%. We found no significant difference between monocular recording and binocular recordings using the McNemar test (OD: *χ*^2^ = 0.24, *P* = 0.63; OS: *χ*^2^ = 0.31, *P* = 0.58).

**Table 1.** Automated RAPD Test Compared Against Post Hoc Impression

		Post Hoc Reference		
		RAPD	Negative	
Automated RAPD test	RAPD	37	4	90.2%
		48.4%	5.2%	positive predictive value
	Negative	8	28	77.8%
		10.4%	36.4%	negative predictive value
		82.2%	87.5%	84.4%
		Sensitivity	Specificity	Accuracy

**Table 2.** Swinging Flashlight Test Versus Post Hoc Impression

		Post Hoc Reference		
		RAPD	Negative	
Swinging flashlight	RAPD	41	5	89.1%
		53.2%	6.3%	Positive predictive value
	Negative	4	27	87.1%
		5.1%	35.4%	negative predictive value
		91.1%	84.4%	88.3%
		Sensitivity	Specificity	Accuracy

We found a significant correlation (r = −0.82, *P* < 0.01) in a subset of 32 patients using Pearson's Linear Correlation Coefficient between our RAPDSize feature and the neutral density filter value used to balance the RAPD in the swinging flashlight examination. The difference in visual acuity in logMAR, between the two eyes correlated significantly (r = −0.65, *P* < 0.01) with the RAPDSize. The difference in the age adjusted MD in the visual field examination, using an Octopus900 perimeter, correlates significantly with the RAPDsize (r = −0.72, *P* < 0.01). All above mentioned correlations are visualized in Figure 3.

The pupillary contraction amplitude was 16.1% bigger in the eye that was stimulated as compared to the contralateral eye. This contraction anisocoria was statistically significant using the two-sample *t*-test (*P* < 0.001).

## Discussion

In a series of 77 recordings of patients (57) and healthy volunteers (20), we found that a commercially purchased hardware designed for consumer electronics allows the reliable determination of the presence of an RAPD within 1 minute.

Our sensitivity (90.2%) and specificity (82.2%) seem higher than reported by Wilhelm et al.^14^ who only correctly detected 85% of RAPDs >0.3logU. This improvement may be due to a superior system, but is more likely because our analysis compares our device to the presence of an optic neuropathy based on multiple clinical findings, as opposed to the clinically measured RAPD, as the gold standard. In our experience, RAPD assessment tends to be unreliable and Wilhelm et al.^14^ may have thus underestimated their device.

Wilhelm et al.^14^ stressed the importance of binocular measurements in their discussion because of contraction anisocoria.^15^ We also found a significant contraction anisocoria in our data. That means the pupillary response from the illuminated eye was about 15% bigger as compared to the contralateral eye. This small difference may, from a theoretical viewpoint, lead to the detection of an RAPD in the eye that is not observed. Practically, however, we found a similar accuracy rate, independent on whether one eye or both eyes were included in the analysis. We conclude that, if possible, both eyes should be used for measurements, but a monocular recording is also sufficient. We found no significant differences between RAPDs measured binocularly and monocularly.

Switching the surveyed eye together with the illumination as is done in the traditional swinging flashlight test led to a worse separation in our case as shown in Supplementary Figure S1. In the post hoc manual examination of the misclassified records, we often noticed a small anisocoria in the pupillography records, which could be the reason for the misinterpretation of the swinging flashlight test. Lam et al.^15^ quantified this influence to be 0.14 log unit of RAPD for each millimeter of anisocoria. Because an automated measure can use either eye for analysis and can use a single pupil to quantify the pupillary response of both eyes, this method is not influenced by anisocoria. Similarly, as with anisocoria, the automated measure may be less sensitive to any variability of pupil size. In patients with hippus, the swinging flashlight test may be impaired by physiological spontaneous changes in pupil size. The averaging of several responses and the possibility to attribute the peak of a pupil response to a light stimulus and compare it to the preceding moment makes the automated procedure less sensitive to such sources of error. The same is possibly true for patients with nystagmus, strabismus, or attention deficit disorders. In these cases, repeated measures may average out possible artifacts. We have not examined such patients in this study and thus cannot draw a conclusion in this regard.

A drawback of the RAPD is that it can only detect relative defects from unilateral pathology or asymmetric optic neuropathy. A measure of the absolute afferent pupillary defect would allow the detection and quantification of bilateral cases and would therefore theoretically be superior. However, the published testing protocols for the absolute APD are time-consuming, the normal range of afferent pupillary defect/non-defect is relatively large, and it is influenced by attention and accommodation.^16^ Due to all these restraints, we focused on the measure of the relative afferent pupillary defect rather than the absolute afferent pupillary deficit.^17^

Our study was limited by the fact that the clinicians in our study were not blinded and had access to past data and all clinical examinations. Given that the clinicians, but not the VR headset, had knowledge about the prior probability of the presence of an RAPD, the accuracy of the clinicians may be overestimated (i.e. the performance of the VR headset in comparison to a clinician may be underestimated). This may have played a role in the frequency of false positive responses. As pointed out by Kawasaki et al., some healthy patients may present with an RAPD.^17^ Indeed, in three out of nine patients, both methods, the manual swinging flashlight test and the automatic measure, demonstrated an RAPD (data not shown). These might be the patients that show a physiological afferent pupillary deficit as described by Kawasaki et al. For the remaining six “false positive” cases measured by the automated procedure, some may actually be “false negative” cases in the swinging flashlight test, as the non-blinded observer may have been predisposed to overlook an RAPD in a patient that was obviously clinically healthy. We cannot further elaborate on this, as this point is outside the scope of the current study.

Another limitation is that we used data of examinations for one particular VR headset. We believe that our technique may readily be used in other VR devices that are capable of recording pupil size. The brightness and darkness of the screen in the FOVE0 is not particularly better than those of any other cell phone screens. The sampling rate, which may be critical for other purposes, is not critical when recording the rather slow pupillary responses. In line with this, currently unpublished data acquired at our laboratory shows that the same procedure is also possible with a different VR headset. An additional limitation is that the accuracy we measured was obtained with a somewhat arbitrary stimulation protocol. Last, we only included pupillary amplitude in our analysis, therefore, it is possible that a variable stimulus duration, variable light intensities, or stimulus color, along with analysis of the pupil velocity and/or escape response, may show even better results than the version we present in the current study.

## Supplementary Material

Supplement 1

## References

[1] Relative Afferent Pupillary Defect - EyeWiki. Accessed September 9, 2022. Available at: https://eyewiki.aao.org/Relative_Afferent_Pupillary_Defect.

[2] Levatin P, Prasloski PF, Collen MF. The swinging flashlight test in multiphasic screening for eye disease. *Can J Ophthalmol*. 1973; 8(2): 356–360. 4350500

[3] Thompson HS. Afferent pupillary defects. Pupillary findings associated with defects of the afferent arm of the pupillary light reflex arc. *Am J Ophthalmol*. 1966; 62(5): 860–873. 5928836

[4] Thompson HS, Corbett JJ, Cox TA. How to measure the relative afferent pupillary defect. *Survey Ophthalmol*. 1981; 26(1): 39–42. 10.1016/0039-6257(81)90124-7 7280994

[5] Boucher T, Fortin É, Evoy F. The standard swinging flashlight test: reliable or not (P1.9-009). *Neurology*. 2019; 92(15 Supplement): P1.9–009.

[6] Hennessy AL, Katz J, Ramakrishnan R, et al. The utility of relative afferent pupillary defect as a screening tool for glaucoma: prospective examination of a large population-based study in a south Indian population. *Br J Ophthalmol*. 2011; 95(9): 1203–1206. 21349935 10.1136/bjo.2010.194217

[7] Lowenstein O. Clinical pupillary symptoms in lesions of the optic nerve, optic chiasm, and optic tract. *AMA Arch Ophthalmol*. 1954; 52(3): 385–403. 13188481 10.1001/archopht.1954.00920050387006

[8] Cohen LM, Rosenberg MA, Tanna AP, Volpe NJ. A novel computerized portable pupillometer detects and quantifies relative afferent pupillary defects. *Curr Eye Res*. 2015; 40(11): 1120–1127. 25658805 10.3109/02713683.2014.980007

[9] Volpe NJ, Plotkin ES, Maguire MG, Hariprasad R, Galetta SL. Portable pupillography of the swinging flashlight test to detect afferent pupillary defects. *Ophthalmology*. 2000; 107(10): 1913–1921; discussion 1922. 11013198 10.1016/s0161-6420(00)00354-7

[10] Riva G, Wiederhold BK. The new dawn of virtual reality in health care: medical simulation and experiential interface. *Stud Health Technol Inform*. 2015; 219: 3–6. 26799868

[11] Adhanom IB, MacNeilage P, Folmer E. Eye tracking in virtual reality: a broad review of applications and challenges [published online ahead of print January 18, 2023]. *Virtual Reality*. 2023; 27: 1481–1505, 10.1007/s10055-022-00738-z. PMC10449001 37621305

[12] World Medical Association (AMM). Declaration of Helsinki. Ethical principles for medical research involving human subjects. *JAMA*. 2013; 310(20): 2191–2194. 24141714 10.1001/jama.2013.281053

[13] McNemar Q. Note on the sampling error of the difference between correlated proportions or percentages. *Psychometrika*. 1947; 12(2): 153–157. 20254758 10.1007/BF02295996

[14] Wilhelm B, Lüdtke H, Peters T, Schmid R, Wilhelm H, Zrenner E. [Automated swinging flashlight test in patients with optic nerve diseases]. *Klin Monbl Augenheilkd*. 2001; 218(1): 21–25. 11225395 10.1055/s-2001-11256

[15] Lam A, Chan R, Lam CH. The validity of a new noncontact tonometer and its comparison with the Goldmann tonometer. *Optomet Vis Sci*. 2004; 81(8): 601–605. 10.1097/01.opx.0000141796.95597.43 15300119

[16] Mathot S . Pupillometry: psychology, physiology, and function. *J Cognition*. 2018; 1(1): 16. 10.5334/joc.18 PMC6634360 31517190

[17] Kawasaki A, Moore P, Kardon RH. Long-term fluctuation of relative afferent pupillary defect in subjects with normal visual function. *Am J Ophthalmol*. 1996; 122(6): 875–882. 8956643 10.1016/s0002-9394(14)70385-x

