# Supplementary material for: Detection of Relative Afferent Pupillary Defects Using Eye Tracking and a VR Headset
Source: Transl Vis Sci Technol. 2023 Jun 27;12(6):22. doi: 10.1167/tvst.12.6.22 (PMC10309159; doi:10.1167/tvst.12.6.22)
Supplement: Supplement 1 [file tvst-12-6-22_s001.pdf]

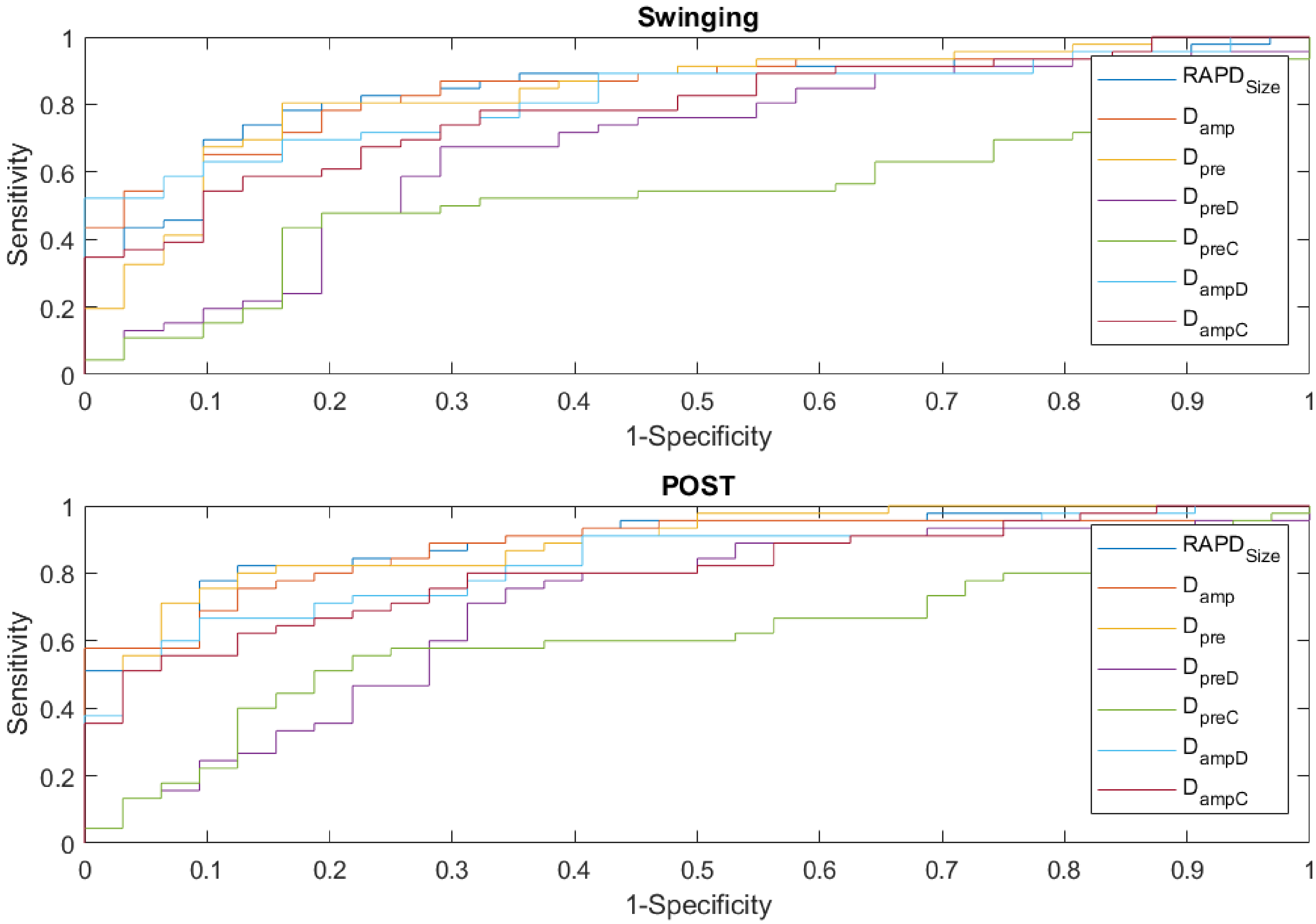

Supplementary Figure 1

Receiver operating characteristic (ROC) for different extracted features. ROC with the post-hoc impression used as reference. Description of the features: RAPD_size binocular feature including prestimulus amplitude differences and contraction amplitude differences; D_amp mean of the

right and left eye of the contraction amplitude difference; D_pre mean of the right and left eye prestimulus amplitude difference; D_preD amplitude difference measured on the illuminated eye; D_preC amplitude difference measured on the not illuminated eye; D_ampD contraction amplitude difference measured on the illuminated eye; D_ampC contraction amplitude difference measured on the not illuminated eye;
